# Press release guide for genomic research and medicine: a framework co-developed with public contributors in Japan

**DOI:** 10.1038/s10038-026-01452-3

**Published:** 2026-01-15

**Authors:** Misaki Arakawa, Tomoyo Takeuchi, Yusuke Ebana, Kaori Muto, Masayuki Yoshida, Fuji Nagami

**Affiliations:** 1 Tohoku Medical Megabank Organization, Tohoku University, Sendai, Miyagi Japan; 2 Tohoku University Graduate School of Medicine, Sendai, Miyagi Japan; 3 Tsukuba Human Tissue Biobank Center, University of Tsukuba Hospital, Tsukuba, Ibaraki Japan; 4 Life Science and Bioethics Research Center, Institute of Science Tokyo, Tokyo, Japan; 5 The Institute of Medical Science, The University of Tokyo, Tokyo, Japan; 6 RIKEN Center for Integrative Medical Sciences, Yokohama, Kanagawa Japan; 7 Tohoku University Advanced Research Center for Innovations in Next-Generation Medicine, Sendai, Miyagi Japan

**Keywords:** Ethics, Genetics research

## Abstract

Press releases on genomic research play an important role in Japan. Not only do journalists use them as major sources of news stories, but the public also accesses them directly across various media platforms. Given the unique characteristics of genomic information, including ethical implications, it is essential to report research results in a responsible and comprehensive manner. To support individuals involved in communicating such results, we developed a press release guide for genomic research in collaboration with diverse stakeholders, including members of the public. This guide outlines 12 key items for scientific research press releases, five of which are tailored to genomic research: protect personal information; avoid detrimental behavior change; consider individuals involved; avoid prejudice or discrimination; and avoid “genetic determinism.” For each item, supplementary explanations and guidance are presented in the form of “Note” and “For Media.” Additionally, fresh insights gained through the discussions with the diverse stakeholders are incorporated into a new section titled “From a different viewpoint.” Genomic research is increasingly integrated into clinical practice and society, which indicates that reports on genomic studies directly affect a broader audience. Furthermore, enhancing the reporting quality of genomic research is critical because of the distinctive characteristics of genomic information. Our guide provides practical support for those disseminating genomic research results and will be valuable in promoting communication among stakeholders with different roles and expectations.

## Introduction

Over the past two decades, genomic research and medicine have developed rapidly, with a dramatic increase in the number of published articles in the field [1, 2]. Since the completion of the Human Genome Project in 2003, there has been an increasing number of media reports concerning human diseases and traits in the context of genomic medicine and research. This trend is particularly evident when public figures attract attention by utilizing genomic advances for medical treatment and other purposes. Journalists rely on various sources for their reporting on medical research, including journal articles, press releases, and information from research institutions or hospitals, with press releases regarded as a particularly useful source [3, 4]. Press releases are official documents to inform the media about research findings. In Japan, they are mainly issued by academic institutions or companies. Our previous study revealed that approximately 77% of news reports on genomic research and medicine in Japan were based on press releases issued by research institutes (Nagami F et al., 2024, unpublished data), indicating that press releases significantly influence subsequent media coverage.

Previous studies on the relationship between press releases and health-related news have demonstrated that the quality of press releases strongly influences the quality of health news stories [5–7]. To improve the quality of press releases, several science and medical communication guides have been developed and made publicly available in various countries [8–11]. A guide specifically tailored to genomic research would need to be made, given the unique characteristics of genetic information compared to other forms of biological or personal data [12, 13]. Its key attributes include its predictive nature, familial relevance, and lifelong stability, but these are not covered in the existing medical communication guides. Furthermore, there is no common framework for preparing press releases, which are a major communication tool connecting genomic researchers with the public. This is despite the consensus among the genetic research community in Japan on the vital importance of promoting public understanding and trust [14].

Another important issue in the current reporting landscape is the public accessibility of press releases online. Because the public is likely to encounter these materials across various media platforms, it is essential that press releases carefully address the ethical implications of genomic information, especially considering the potential impact on readers directly affected by the reported study results. To ensure that genomic research results are reported ethically, researchers and press officers must adopt broader perspectives that reflect the diverse viewpoints of patients and the public. A method for incorporating diverse perspectives into research process, known as patient and public involvement or public engagement, has been identified as a significant aspect of research practice [15, 16] and has gained increasing recognition in Japan. This approach also holds considerable promise for improving how genomic research is communicated through press releases.

This article introduces a press release guide for genomic research, which was developed by incorporating the perspectives of patients and the public into each item. Considering the possibility that genomic press releases have a direct and significant impact on readers, a standardized, agreed-upon framework for disseminating research results would benefit all stakeholders involved. In addition to presenting the guide, this article focuses on the significant points to consider when reporting genomic research results, which would also facilitate communication among individuals with different roles and expectations.

## Press release guide for genomic research and medicine

**Fig. 1:**
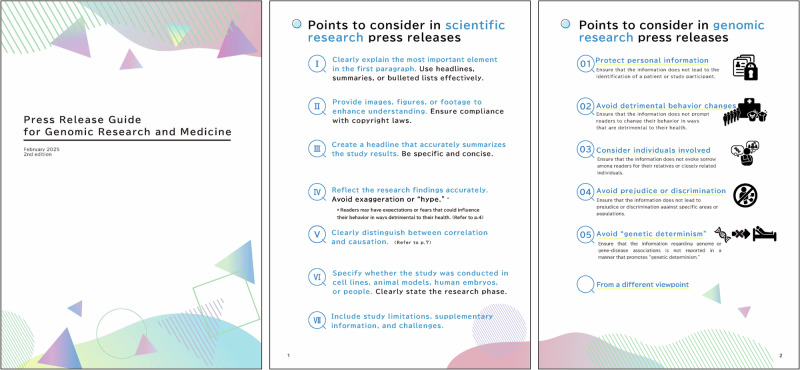
Excerpts from the 9-page guide. The cover of the guide (left) and an overview of the twelve key points to consider (center and right), including five points specific to genomic research press releases, each represented by a newly designed pictogram

We released a Press Release Guide for Genomic Research and Medicine in Japan in October 2024. The guide comprises two areas: scientific and genomic research press release consideration (Fig. 1). The first scientific research section contains seven items drawn from three existing guides, which target medical researchers and press officers (Table 1: A, B, and E). The seven suggestions, included in each of the reference guides, were considered essential to genomic research, and therefore, incorporated into our guide. All suggestions from guides A, B, and E are included in Supplementary Information 1.

**Table 1:** Existing press release guides for medical and science research

	Title	Country	Target	Selected for reference
A	Guide to Disseminate Medical Research Results in an Easy-to-Understand Manner [2nd edition]*1	Japan	medical researchers/press officers	Yes
B	Japan Neuroscience Society (JNS) Science Communication Guideline for Research Results Press Releases*2	Japan	medical researchers/press officers	Yes
C	The Japanese Psychological Association (JPA) Science Communication Guideline for Research Results Press Releases*3	Japan	medical researchers/press officers	No (approximately same as B)
D	Media Doctor (review criteria)*4	Australia, Canada, Germany, Japan, United States	medical journalists	No (different target)
E	Stempra Guide to being a Media Officer [3rd edition] *5	United Kingdom	science press officers	Yes
F	PR-Rx: Press Release Reporting Exemplar [EQUATOR]*6	United Kingdom	medical researchers/press officers/journalists	No (under development)

Using publicly accessible search engines (Google Scholar and Google), these six guides or criteria related to medical and scientific research were identified

*1) https://ez2understand.ifi.u-tokyo.ac.jp/library/guidebook/

*2) https://www.jnss.org/hp_images/images/Science%20Communication%20Guidelines.pdf

*3) https://psych.or.jp/wp-content/uploads/2020/04/SC_guideline.pdf

*4) https://mediadoctor.jp/menu/review.html

*5) https://stempra.org.uk/

*6) https://www.equator-network.org/library/reporting-guidelines-under-development/reporting-guidelines-under-development-for-other-study-designs/#PRRX

**Fig. 2:**
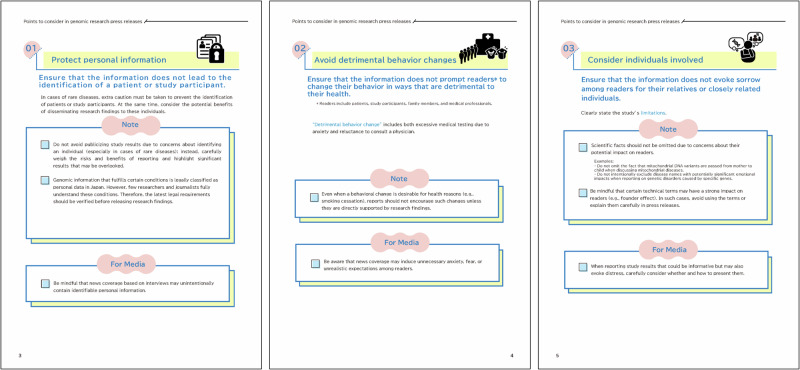
Excerpts from the 9-page guide. Items No. 1 (left), 2 (center), and 3 (right). Each key item includes additional sections titled “Note” and “For Media”

**Fig. 3:**
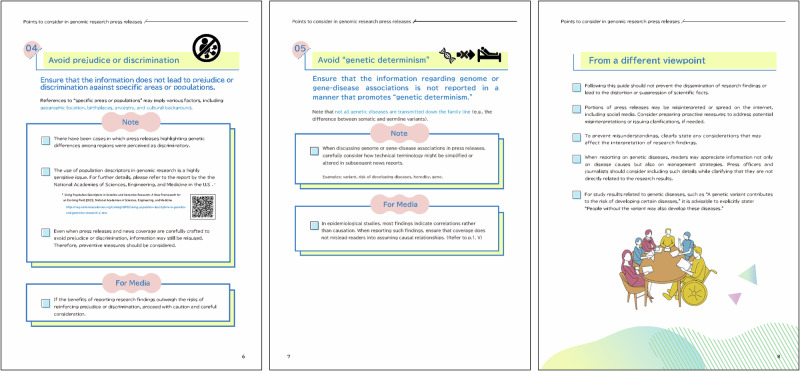
Excerpts from the 9-page guide. Items No. 4 (left), 5(center), and an additional page, which presents the “From a different viewpoint” section (right)

The second genomic section includes five items tailored to genomic research press releases. These items are based on a literature review on the ethical, legal, and social issues in genomic research, and we identified the distinctive characteristics of genomic information that should be carefully considered in press releases. For example, genomic study results may inadvertently identify specific patients [17, 18]. Even with anonymization and data protection, individuals with rare genetic diseases may still be identifiable owing to small patient populations. Moreover, certain findings may stigmatize groups—such as minorities or those with genetic predispositions to psychiatric conditions—who may fear resulting discrimination [17]. Media reports may also misrepresent gene–disease associations with headlines like “discovery of the genetic variant that causes ‘disease A’!” even when the variant is only one of multiple contributing factors [17, 19]. Such reporting can influence public perceptions and behaviors. On the basis of this review and subsequent discussions with public contributors, we formulated five original items specific to genomic research. The highlight of this section is that supplementary explanations for each item, as well as the broader context, are presented in the form of a “Note,” and tailored guidance for media professionals is labeled “For Media” (Fig. 2). Furthermore, fresh insights gained through the discussions are incorporated into a new section titled “From a different viewpoint,” which consists of significant issues that had previously been overlooked in the communication of study results (Fig. 3). The twelve items along with detailed explanations of the five genomic items are as follows.

### Seven points to consider in scientific research press releases

1. Clearly explain the most important element in the first paragraph. Use headlines, summaries, or bulleted lists effectively.
2. Provide images, figures, or footage to enhance understanding. Ensure compliance with copyright laws.
3. Create a headline that accurately summarizes the study results. Be specific and concise.
4. Reflect the research findings accurately. Avoid exaggeration or “hype.” *
  *Readers may have expectations or fears that could influence their behavior in ways detrimental to their health.
5. Clearly distinguish between correlation and causation.
6. Specify whether the study was conducted in cell lines, animal models, human embryos, or people. Clearly state the research phase.
7. Include study limitations, supplementary information, and challenges.

### Five points to consider in genomic research press releases

#### [No.1: Protect personal information]

Ensure that the information does not lead to the identification of a patient or study participant.

In cases of rare diseases, extra caution must be taken to prevent the identification of patients or study participants. At the same time, consider the potential benefits of disseminating research findings to these individuals.

(Note 1) Do not avoid publicizing study results due to concerns about identifying an individual (especially in cases of rare diseases); instead, carefully weigh the risks and benefits of reporting and highlight significant results that may be overlooked.

(Note 2) Genomic information that fulfills certain conditions is legally classified as personal data in Japan. However, few researchers and journalists fully understand these conditions. Therefore, the latest legal requirements should be verified before releasing research findings.

Even with anonymization, individuals with rare genetic diseases may still be identifiable, and thus, their data need to be strictly protected. Data protection could conflict with the goal of publicizing study results widely. Item No. 1 focuses on the conflicting elements of reporting results. Discussions with public contributors enabled us to conclude that carefully weighing the risks and benefits of reporting is more important than avoiding publicizing study results because of concerns about identifying an individual. Additionally, lesser-known legal requirements for genomic information are indicated with a QR code for the related guidelines’ website.

#### [No.2: Avoid detrimental behavior changes]

Ensure that the information does not prompt readers* to change their behavior in ways that are detrimental to their health (*readers include patients, study participants, family members, and medical professionals).

“Detrimental behavior change” includes both excessive medical testing due to anxiety and reluctance to consult a physician.

(Note) Even when a behavioral change is desirable for health reasons (e.g., smoking cessation), reports should not encourage such changes unless they are directly supported by research findings.

(For Media) Be aware that news coverage may induce unnecessary anxiety, fear, or unrealistic expectations among readers.

Reporting a novel finding of a pathogenic variant may potentially result in behavioral changes in a broader range of people in ways that could prove harmful to their health. This item is meant to remind people in charge of reporting that it is essential to be mindful of the influence of certain information or expressions on readers. The “readers” in this item include medical professionals, because we want them to avoid detrimental behavior changes that have been associated with knowledge gained from media coverage.

#### [No.3: Consider individuals involved]

Ensure that the information does not evoke sorrow among readers for their relatives or closely related individuals.

Clearly state the study’s limitations.

(Note 1) Scientific facts should not be omitted due to concerns about their potential impact on readers.

Examples:

-Do not omit the fact that mitochondrial DNA variants are passed from mother to child when discussing mitochondrial diseases.

-Do not intentionally exclude disease names with potentially significant emotional impacts when reporting on genetic disorders caused by specific genes.

(Note 2) Be mindful that certain technical terms may have a strong impact on readers (e.g., founder effect). In such cases, avoid using the terms or explain them carefully in press releases.

Similar to No.2, this item is also meant to consider the impact on readers. Some information, such as about diseases caused by pathogenic variants that are inherited from parents, may evoke sorrow or distress among people who have the variants. To avoid this, we have added practical suggestions, such as clearly stating the study’s limitations and how to deal with certain technical terms that may have a strong impact on readers. At the same time, we emphasize the importance of publicizing scientific facts, as urged by contributors.

#### [No.4: Avoid prejudice or discrimination]

Ensure that the information does not lead to prejudice or discrimination against specific areas or populations.

References to “specific areas or populations” may imply various factors, including geographic location, birthplaces, ancestry, and cultural background.

(Note 1) There have been cases in which press releases highlighting genetic differences among regions were perceived as discriminatory.

(Note 2) The use of population descriptors in genomic research is a highly sensitive issue. For further details, please refer to the report by the National Academies of Sciences, Engineering, and Medicine in the U.S.

(Note 3) Even when press releases and news coverage are carefully crafted to avoid prejudice or discrimination, information may still be misused. Therefore, preventive measures should be considered.

We incorporated Notes 1 and 3, which were based on opinions from experienced press officers, as a reminder that scientific facts on genetic differences may be misused in discriminatory ways, especially in this era of social media. To deal with this problem, preventive measures should be considered in advance. Regarding Note 2, the use of population descriptors in genomic research, such as “race” or “ethnicity,” has been debated internationally. We introduce a report released by the National Academies of Sciences, Engineering, and Medicine [20], with a QR code for the report.

#### [No.5: Avoid “genetic determinism”]

Ensure that the information regarding genome or gene-disease associations is not reported in a manner that promotes “genetic determinism.”

Note that not all genetic diseases are transmitted down the family line (e.g., the difference between somatic and germline variants).

(Note) When discussing genome or gene-disease associations in press releases, carefully consider how technical terminology might be simplified or altered in subsequent news reports.

Examples: variant, risk of developing diseases, heredity, gene.

(For Media) In epidemiological studies, most findings indicate correlations rather than causation. When reporting such findings, ensure that coverage does not mislead readers into assuming causal relationships.

The public tend to view the genome as a decisive factor in determining a person’s life, which is known as “genetic determinism.” This trend has shifted recently with the advancement of genomic medicine, but media are still influenced by the deterministic viewpoint. To avoid the misrepresentation of gene–disease associations, we have added supplementary explanations of genetic disease origin and technical terminologies to this item.

### From a different viewpoint

- Following this guide should not prevent the dissemination of research findings or lead to the distortion or suppression of scientific facts.
- Portions of press releases may be misinterpreted or spread on the internet, including social media. Consider preparing proactive measures to address potential misinterpretations or issuing clarifications, if needed.
- To prevent misunderstandings, clearly state any considerations that may affect the interpretation of research findings.
- When reporting on genetic diseases, readers may appreciate information not only on disease causes but also on management strategies. Press officers and journalists should consider including such details while clarifying that they are not directly related to the research results.
- For study results related to genetic diseases, such as “A genetic variant contributes to the risk of developing certain diseases,” it is advisable to explicitly state: “People without the variant may also develop these diseases.”

## Significant role in the media landscape and society

To the best of our knowledge, this is the first open-access press release guide specifically developed for genomic research. It is intended for all individuals involved in writing or disseminating press releases and related news coverage in this field.

Previous research investigating the influence of press releases on the quality of health-related science news has found that high-quality press releases tend to improve the quality of related newspaper stories, and vice-versa [5–7]. Some studies identified exaggeration or the absence of caveats in press releases as contributing factors to inaccurate news coverage and suggested that improving press release accuracy may help reduce misleading health news [5, 6]. Another study revealed interrelated factors that promote hype in genomic research, identifying press releases as either sources of hype or points of distortion [21]. To improve the quality of press releases and media reporting in science and medicine, several guides have been developed and publicly disseminated in Japan and internationally. The “Guide to Disseminate Medical Research Results in an Easy-to-Understand Manner,” which includes glossaries and a checklist of 20 key considerations for researchers and press officers, has been actively promoted to encourage effective use [8]. The “Japan Neuroscience Society (JNS) Science Communication Guideline for Research Results Press Releases” offers field-specific considerations tailored to neuroscience [9]. The “Stempra Guide to being a Media Officer” provides general guidance for press officers along with checklists specific to health and biomedical research [10]. Our guide shares the same aim as these existing resources and also targets researchers and press officers responsible for disseminating findings in health-related science.

A distinctive feature of genomic research is its growing integration into biomedical science, clinical practice, and society [22]. This indicates that genomic research reports have had a direct impact on a wider range of people. Consequently, reports on genomic studies directly impact a broader audience. Exaggerations or misinterpretations in press releases may lead to more serious behavioral changes among readers. By incorporating perspectives from stakeholders with diverse roles, our press release guide is intended to help mitigate such risks when disseminating information on genomic research.

## Unique features of this guide

### Development with various stakeholders and the public

A notable feature of this guide is that it was developed by respecting suggestions and insights from various stakeholders and the public. To obtain broader opinions on the draft five items and the social impact of press releases related to genomic research, we conducted focus group interviews (FGIs) and group discussions (GDs). The FGIs were conducted with people who had experience contributing to press releases on genomic research. After the FGIs, we conducted GDs with our research team, which included patients and members of the public. (The participants’ characteristics as well as details of the FGIs and GDs can be found in Supplementary information 2.) The participants’ opinions improved and enriched the guide’s content. Another significant implication of this study is that discussions among stakeholders with different roles fostered mutual understanding. These exchanges demonstrated that such opportunities can serve as a means to improve genetic and genomic literacy among individuals who are not familiar with the field.

### Catalyst to promote communication among people involved

Another promising function of the guide is its potential to foster communication among those involved in genomic research and medicine. When introducing this guide to academic communities in Japan and abroad, we received a wide range of suggestions and perspectives from participants. The feedback in Japan highlighted the importance of word choice in press releases, as the language used by researchers and media directly influences public understanding. Open and regular dialogue between researchers and journalists has been proposed to bridge gaps between stakeholders and facilitate mutual understanding. The feedback indicated that the guide itself could function as a catalyst to promote communication among individuals with different roles and expectations. Furthermore, the guide caught the attention of Stempra, a science communication organization based in the United Kingdom, which introduced it on their website. This indicates that similar communication challenges are faced in many countries and that the guide is relevant beyond Japan. However, for its international use, it is important to recognize the differences in communication practices and cultural contexts. We hope that this guide will drive positive changes in the media landscape, with adjustments to fit each country’s context.

## Dissemination, and next steps ahead

We presented the guide at several academic meetings on genetics and genomics in Japan and the United States, where we received feedback from participants. A print version of the guide was distributed by mail to stakeholders, and a PDF version (Japanese/English) was published on our research group’s website: https://genomeppi.jp/topics/20250219.php. (The PDF version is included in Supplementary information 3.) We have not yet widely disseminated the guide, and the limited feedback received indicates room for further refinement in future studies. Furthermore, the guide does not yet address considerations regarding the use of figures or images, which may have significant visual impact and should be addressed in future studies.

In conclusion, improving the reporting quality of genomic research is critical due to the distinctive characteristics of genomic information and the strong influence of media on a public that accesses content across various platforms. Our press release guide provides practical support for those responsible for communicating research results and encourages responsible reporting practices. We expect this guide to improve the quality of press releases and anticipate that it will serve as a standard checklist or protocol in the future. Insights from public contributors also help bridge the divide between researchers and the public. As genomic research continues to evolve and influence diverse areas of medicine, the challenges addressed in this guide will also shift. It is essential to continue disseminating and evaluating the guide while revising its content to reflect developments in the field.

## Supplementary information

Supplementary information1. All suggestions of the three existing guides

Supplementary information2. Details of the guide's development process

Supplementary information3. Press Release Guide for Genomic Research and Medicine (Japanese)
