# Supplementary material for: Press release guide for genomic research and medicine: a framework co-developed with public contributors in Japan
Source: J Hum Genet. 2026 Jan 15;71(3):119–24. doi: 10.1038/s10038-026-01452-3 (PMC12948661; doi:10.1038/s10038-026-01452-3)
Supplement: Supplementary file 1 — Supplementary information1. All suggestions of the three existing guides [file 10038_2026_1452_MOESM1_ESM.pdf]

Supplementary information 1: All Suggestions of the Three Existing Guides

Category	Suggestions	Guides		
		A	B	E
Structure	**Background and aim of making the guide**	**1**	**1**	**1**
	Authors, who understand the research best, should play the chief role in press releases.		1	
	Check whether difficult terms (e.g., medical terms) or abbreviations are included or not. A Glossary should be provided for technical terms.	1	1	
	**Explain the most important element in the first paragraph clearly. Use a headline, summary, or bulleted lists effectively.**	**1**	**1**	**1**
	Provide contact details, including out of hours.		1	1
	Include quotes in the first few paragraphs. (e.g., quotes from a co-author at another university, a funder, a patient group, etc.)			1
Readability	Explain in an easy-to-understand way. (e.g., use active voice and affirmative sentence, avoid making a sentence too long, etc.)	1	1	
	Make the layout clear and easy to read; check text size, space between lines, blank space, etc.	1	1	
	Avoid statistical concepts that are hard to interpret; explain numerical data in an easy-to-understand way.	1		1
	**Provide images, figures, footage, conceptual diagrams, etc. for better understanding; be wary of copyright infringement.**	**1**	**1**	**1**
	Avoid images or figures that could make readers feel unpleasant; if needed, add a note of caution.		1	
	It is desirable to remake figure legends, conceptual diagrams, etc. in Japanese instead of using original ones.		1	
Publication	It is desirable to distribute reference materials to media or post them on websites of research institutes related to the study.		1	
	It is desirable to ask permission of news media to check their story prior to publication.		1	
	If a news article needs corrections or supplementary explanation, it is desirable to post the corrections on the websites of research institutes.		1	
	Decide whether the press release should be issued under embargo or for immediate release. State the date and time clearly.			1
	If JNS science communication committee find a questionable article on neuroscience, the committee may publish comments.		1	
Statistics	It is desirable to include effect size, as well as sample size and p-values.		1	
	**Be clear about whether the reported finding is a correlation or causation.**	**1**	**1**	**1**
	Include, where available, absolute risk as well as relative risk.			1
Research Content	Fully examine whether the images or experimental data to be published will not violate privacy.		1	
	Be diligently checked for accuracy by the original researcher.		1	1
	Be checked by people outside the field (e.g., friends, family members, etc.) after the original researcher's check.	1		
	**State if the study was done in cell lines, animal models, human embryos or in people. State clearly which phase the research is in.**	**1**	**1**	**1**
	State clearly which type of study was undertaken. (e.g., clinical trial, randomized, controlled, double blind, observational study, etc.)	1		1
	Be explicit about whether the press release is about peer-reviewed evidence being published, a conference presentation of unpublished data, etc.			1
	Provide a link to the original study so that readers can refer to it.		1	1
	**Include limitations, supplementary information and challenges of the study.**	**1**	**1**	**1**
	Refer to the academic significance of the research findings.		1	
	Include information that ensures reliability of the research. (e.g., information on IRB/IEC approval, patient case studies if appropriate, etc.)	1		1
	State advantages and disadvantages of the treatment or medicine clearly. Include other options that patient may have.	1		
	Check the amount of information and avoid information overload.	1		
	Be clear about whether it is direct findings of the study or interpretation or extrapolation.		1	1
	Include the background/context of the research. Check it carefully to prevent HARKing (Hypothesizing After the Results are Known).		1	1
	For a conference presentation, provide detailed information on methods and results.		1	
	**Reflect the findings of the research accurately. Avoid exaggerations or 'hype.'**	**1**	**1**	**1**
	**Create a headline that clearly summarizes the study results; be specific and concise.**	**1**	**1**	**1**
	Provide information on research funding and conflict of interest.	1	1	
	Include stories behind the study. (e.g., difficulties experienced by the researchers)		1	

Guide A: Guide to Disseminate Medical Research Results in an Easy-to-Understand Manner [2nd edition]
Guide B: Japan Neuroscience Society (JNS) Science Communication Guideline for Research Results Press Releases
Guide E: Stempra Guide to being a Media Officer [3rd edition]

Scope of the scrutiny
Guide A: pp.5-16; Guide B: all pages; Guide E: pp.11-12, pp.14-15
