# Supplementary material for: Press release guide for genomic research and medicine: a framework co-developed with public contributors in Japan
Source: J Hum Genet. 2026 Jan 15;71(3):119–24. doi: 10.1038/s10038-026-01452-3 (PMC12948661; doi:10.1038/s10038-026-01452-3)
Supplement: Supplementary file 2 — Supplementary information2. Details of the guide's development process [file 10038_2026_1452_MOESM2_ESM.pdf]

Supplementary information 2. Details of the guide's development process

1. Overview of the study

This study involved two sequential steps: Step 1 – development of a draft press release guide based on a literature review and analysis of existing guides; and Step 2 – revision of the draft guide by involving stakeholders and public contributors, which included focus group interviews and group discussions. The study was registered and reviewed by the Tohoku University Tohoku Medical Megabank Organization's Institutional Review Board (2023-4-062).

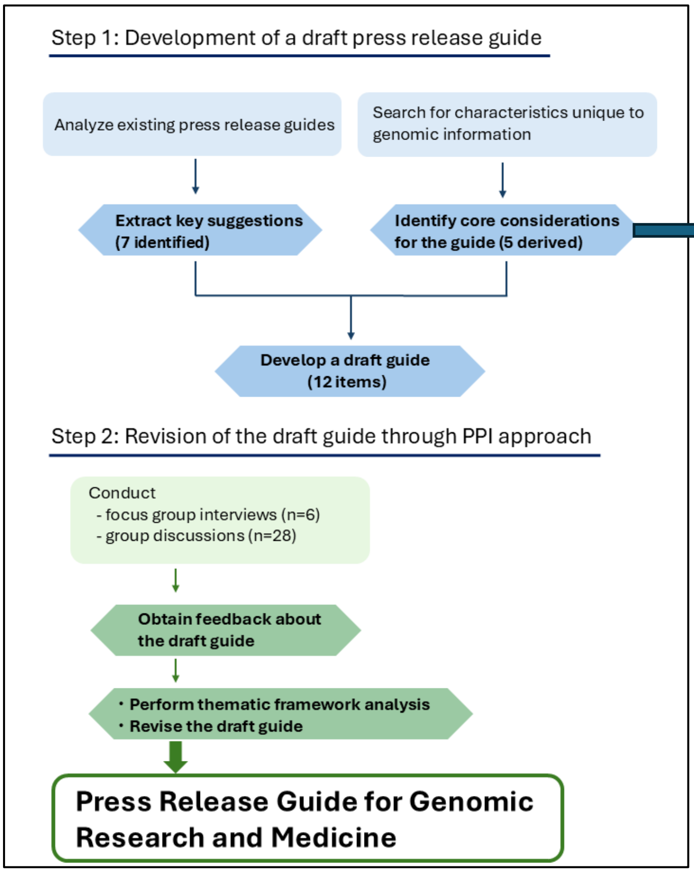

【Draft】Five considerations specific to genomic research

[No.1] Check whether the information could lead to the identification of an individual. (e.g., in rare disease cases)

[No.2] Check whether the information could change the reader's behavior, which may be unnecessary for them.

[No.3] Check whether the information could make genetically related people feel guilty.

[No.4] Check whether the information could place a stigma on certain populations and lead to discrimination against them.

[No.5] Check whether gene-disease associations are reported in a "genetic determinism" way.

To refine the five considerations specific to genomic research, we employed two methods: focus group interviews (FGIs) and group discussions (GDs).

2. Focus group interviews (FGIs) and group discussions (GDs)

【FGIs】Using an interview guide, two researchers (FN and MA) conducted semi-structured interviews on August 3 and 9, 2023, with two focus groups. Each group included a press officer from a research institute, a genomic research scientist, and a journalist in Japan. Participants received three types of press releases on genomic research articles published in April 2022, July 2022, and July 2023 respectively, related news coverage, and the draft press release guide we developed. Each interview lasted approximately two hours, was audio-recorded with prior permission, and transcribed verbatim.

【GDs】Twenty-eight research team members participated in online group discussions on December 12, 2023. We publicly recruited collaborators and selected six of nine applicants to join our research team in March 2023. Among those selected, four were men and two were women, ranging in age from their 20s to 60s. Two individuals had family members affected by diseases (cancer and allergic diseases) and one person had experience of serving on a research ethics committee. The participants' characteristics are described below. The participants received a press release on a genomic research article published in March 2023, related news coverage, and the draft guide. After one researcher (FN) introduced the materials, participants were divided into four diverse groups, each comprising people of various social backgrounds, for 30-minute discussions. Each group then shared its feedback, followed by a collective discussion lasting approximately 20 minutes. With prior consent, the discussions were recorded and transcribed.

**Focus Gourp Interview (FGI)**				**n=6**
	**Occupation**	**Years of experience**	**Workplace setting**	
FGI1	Press officer	10	genomic research institute	1
	Researcher on genomic research	21	genomic research institute	1
	Journalist	20	television station, media agency	1
FGI2	Press officer	20	research institute, university hospital	1
	Researcher on genomic research	23	genomic research institute	1
	Journalist	15	news media organization	1
**Group Discussion (GD)**				**n=28**
	**Occupation**			
	Researcher (Physician)			14(4)
	Patient and the public			6
	Certified Genetic Counselor			4
	Journalist			2
	Research supporter			2

3. Data analysis

The large volume of FGI data was summarized using four steps (unit determination, paraphrasing, generalization, and reduction) based on Mayring's method [1]. Along with the summarized FGI data, GD data were coded using NVivo software and analyzed using thematic framework analysis, following Ritchie and Spencer [2]. The first step was "familiarization" of the data, in which one researcher (MA) independently reviewed the FGI and GD transcripts. The next step was "devising a conceptual framework." This was done by MA, reviewed by another researcher (FN) and refined through discussion. MA then applied the framework to the FGI/GD data ("indexing"). Finally, the indexed data were reviewed by FN and refined through discussion, which distilled the essence of evidence ("sorting and synthesizing").

4. Themes identified in the thematic framework analysis

The conceptual framework was developed based on the FGI interview guide and comprised seven themes: five themes on the genomic-specific items, one on feedback for all items of the draft guide, and one on other issues, including impressions of the guide. Through the subsequent steps of indexing, sorting, and synthesizing, four main themes were identified: (1) terminology and phrasing; (2) ambiguity regarding intended audiences; (3) lack of contextual detail; and (4) risk of misrepresenting scientific facts.

Identified themes, analysis, and participants' quotes

Themes	Analysis and Supporting quotes
**Suggestions about the terms and phrasing**	Several expressions in the draft—such as "unnecessary behavior," "genetically related people," "feel guilty," and "stigma"—were flagged as ambiguous or potentially misleading. Participants noted that "feel guilty" is overly broad and may carry unintended religious connotations.<br><br>*"(Regarding [No.3]) 'Feel guilty' is an ambiguous expression, and I think it difficult to guess what could make people feel guilty without ethical criteria for judging."* (a press officer)<br><br>*"(Regarding [No.3]) "'Genetically related people'" probably indicates consanguine family, but those are not only people who have feelings of guilt."* (a researcher)
**Ambiguity regarding intended audiences**	Participants examined who the target audience was for each item. Terms such as "unnecessary behavior" were considered subjective, as interpretations may vary.<br><br>*"(Regarding all items) The targets of all items are unclear. Do they target the public or patients with genetic diseases? The items' meaning could vary depending on who reads them, so the target s need to be discussed and clarified."* (a genetic counselor)<br><br>*"(Regarding [No.2]) When something is regarded as 'unnecessary,' for whom is it? Even if medical staff judge something to be unnecessary, patients might judge it necessary. What 'unnecessary behavior' indicates is unclear."* (a member of the public)

**Insufficiency of supplementary information**	Most participants indicated that the five items alone were insufficient for readers to accurately understand the intended meaning, and that supplementary information should be included. They suggested that press releases should incorporate practical measures for readers alongside the study results to help alleviate anxiety or improve quality of life. Participants also recommended the inclusion of definitions for technical or potentially confusing genomic terminology that could significantly impact readers.<br><br>*"(Regarding [No.3]) I want a caveat to be included in a press release, such as 'Many studies in this field have found results related to this study, and our result is one of them' or 'This is a viewpoint of the researchers.'"* (a journalist)<br><br>*"(Regarding all items) If a press release mentions that certain genetic variants are associated with an increased risk of some diseases, I want the opposite possibility to be also included (i.e., people without the variants could also have the diseases)."* (a member of the public)
**Concerns regarding potential distortion of scientific facts**	Several participants expressed concerns that excessive caution could result in distortion or omission of scientific facts. While there was strong consensus on the importance of protecting personal information, participants also emphasized the value of disseminating research findings, especially those that could benefit patients with rare diseases.<br><br>*"(Regarding all items) I don't want researchers to hesitate to report research results because of their higher priority on protecting personal information."* (a journalist)<br><br>*"(Regarding [No.1]) Regarding clinical trial results of rare diseases, I always wonder whether press releases should be issued, because of the fewer number of participants. But I also think it is a responsibility of researchers to report their research results to the participants."* (a press officer)

1. P. Mayring. Qualitative content analysis: theoretical foundation, basic procedures and software solution. Klagenfurt. Available at Social Science Open Access Repository (SSOAR). 2014 [Accessed September 15, 2023] Available from: https://www.ssoar.info/ssoar/bitstream/handle/document/39517/ssoar-2014-mayring-Qualitative_content_analysis_theoretical_foundation.pdf

2. J. Ritchie, L. Spencer, and W. O'Connor. Carrying out qualitative analysis. In: Edited by J. Ritchie and J. Lewis. Qualitative research practice: A guide for social science students and researchers. London: Sage 2003. P. 219-262.
