# Supplementary material for: Press release guide for genomic research and medicine: a framework co-developed with public contributors in Japan
Source: J Hum Genet. 2026 Jan 15;71(3):119–24. doi: 10.1038/s10038-026-01452-3 (PMC12948661; doi:10.1038/s10038-026-01452-3)
Supplement: Supplementary file 3 — Supplementary information3. Press Release Guide for Genomic Research and Medicine (Japanese) [file 10038_2026_1452_MOESM3_ESM.pdf]

ゲノム医療・研究の プレスリリース発出ガイド

2025年2月　第2版

序文

　ゲノム医療・研究における患者・市民参画のプロジェクトの中で、リテラシーや情報発信の問題を扱えないか。今回、この「ゲノム医療・研究のプレスリリース発出ガイド」を作る出発点となった問題意識でした。参画（involvement）は、医療や研究のさまざまなシーンで、患者や市民の方々がその意思を表明したり反映させたりすることですが、そもそもどのくらい知識や理解をもって臨んでいるか、といったリテラシーの状況や、医療・研究の側からの情報発信の状況を踏まえなければ、砂上の楼閣になるのではないか、と考えたためです。研究者にとって、研究成果の情報発信やコミュニケーションの手段としてプレスリリースが重視されていることを確かめ、また、新聞報道における記事もそのプレスリリースに基づいたものが多いことを明らかにした上で、このガイドの制作を進めてきました。

　ガイド制作を志したプロジェクトは、日本医療研究開発機構（AMED）のゲノム医療実現バイオバンク利活用プログラム令和4年度採択課題「ゲノム医療・研究推進社会に向けた試料・情報の利活用とPPI施策に関する研究開発」（代表：吉田雅幸 東京科学大学教授）です。この課題を共に進めている多くの方々には本制作にあたってひとかたならぬお世話になりました。また、ガイドの制作を進める過程では、先行して類似の取組をされていた、医療情報をわかりやすく発信するプロジェクトや、日本神経科学学会、英国の科学広報担当者の集まりであるSTEMPRAの事例に学ぶと共に、インタビュー調査に参加いただいた沢山の方々、グループディスカッションに参加いただいた一般の方々を含む多様な方々に、大変お世話になりました、ここに改めて御礼申し上げます。

　本ガイドは、ゲノム医療・研究とその情報発信、そして受信に関わる方々と共有するための、あくまでの試案として使われ、批判を受け、修正され、補強されて完成されていくものと考えています。ゲノム医療・研究の情報発信の場で、多く参照いただくと共に、たくさんのフィードバックをいただけることを願ってやみません。

制作者を代表して
　東北大学東北メディカル･メガバンク機構
　教授　長神風二

目次

序文・・・1

目次・・・2

プレスリリースとは・・・3

研究成果プレスリリースの留意事項
～科学の分野すべてで共通すること～・・・4

ゲノム医療・研究のプレスリリース発出ガイド・・・5

参考文献・・・12

プレスリリースとは

“ニュース”にしてもらうために公式文書を出すこと

ゲノム医療・研究の場合には、
- 大学・研究所・企業などが報道機関向けに発出
- Ａ４の紙に３‐７枚程度のものが多く、新しい発見についてなど、その概要を図入りで説明するものが多い
- 記者会などに文書で配布されると共に、公式ウェブサイトで公開され、長く掲載され続ける
- プレスリリースの発出と同時に、説明会（記者会見）が開かれることがある

学術的な定義

「(ニュースリリースともいう。) 企業や自治体などの組織が、主に報道関係者に向けて情報を提供するための公式な報道発表。(中略) 記者クラブを通じて発表される場合、記者クラブに設置されたメディア各社のボックスに資料が配布される。大きな報道が期待される発表の場合は、組織の上層部が出席する**記者会見**が行われる。レクチャーつきといわれる広報担当者による口頭の説明がついた資料配布などもある。」

(「広報・ＰＲ論パブリック・リレーションズの理論と実際」関谷直也ほか著 有斐閣２０１４年)

プレスリリースと情報の流れ

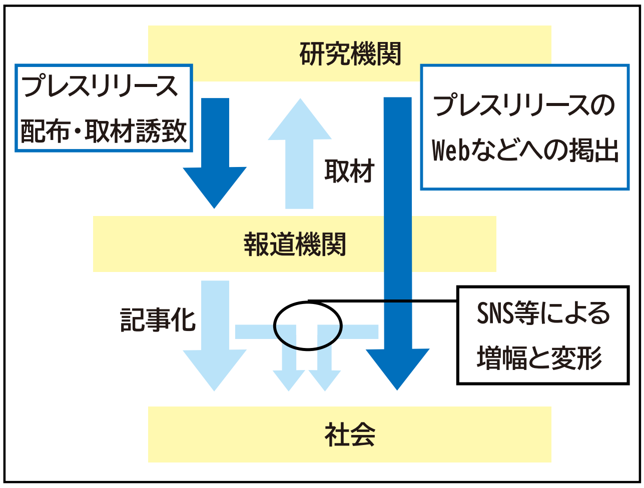

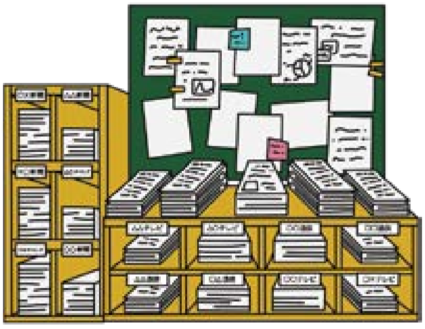

プレスリリース文書の例

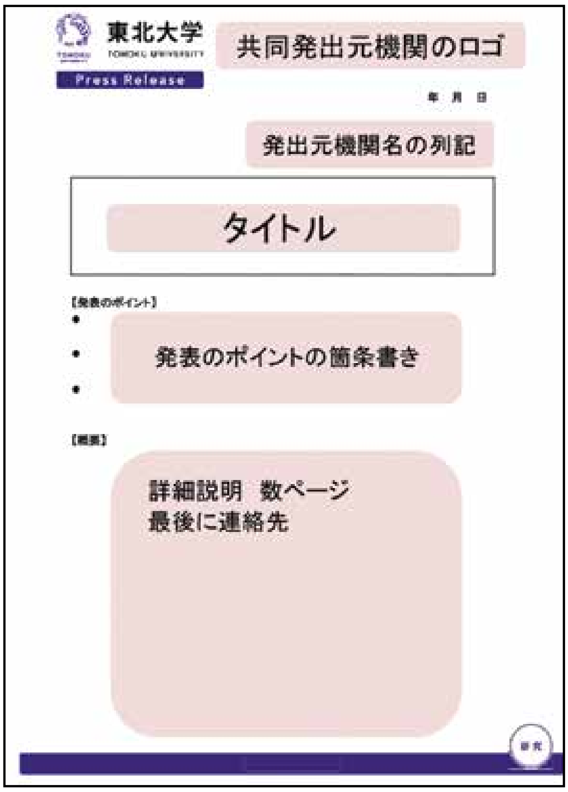

研究成果プレスリリースの留意事項

～科学の分野すべてで共通すること～

Ⅰ 重要な情報を最初に記述し、見出しや箇条書きも活用して、読みやすい資料にする

Ⅱ 写真や図、画像、映像を用いて視覚的にわかりやすい資料にする（著作権に注意）

Ⅲ 誤解を生まないわかりやすいタイトルにする（研究目的を明確に記載）

Ⅳ 研究成果を正確に反映し、大げさな表現や著しい飛躍をしない*

*読んだ人が過度な期待・不安・恐怖などを抱き、不適切な対応をしてしまう可能性に留意すること（P.7と関連）

Ⅴ 相関関係に過ぎないものを因果関係として記載しない*

*P.10と関連

Ⅵ 何を用いたか（細胞株か、動物か、ヒトか）を記載し、研究の進捗段階を明示する

Ⅶ 当該研究の課題や限界、補足を記載する（不確かさを明示）

ゲノム医療・研究のプレスリリース発出ガイド

01 個人情報を守る

患者・研究参加者の個人情報が特定されないような記載にする

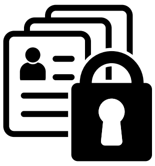

02 不適切な対応等を防ぐ

読んだ人が不適切な対応を行うことがないような記載にする

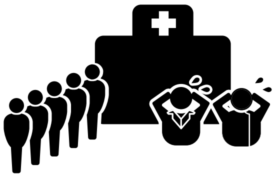

03 関係者に配慮する

患者・研究参加者自身が、またはその親族や近しい人々がお互いに対して、負い目を感じないような記載にする

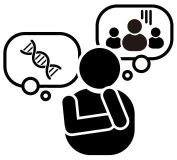

04 偏見・差別を防ぐ

特定の地域や集団への偏見や差別につながらないような記載にする

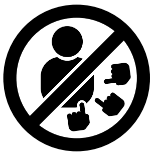

05 遺伝子決定論を避ける

ゲノム・遺伝子と病気との関係について、「特定の違いがあれば必ず病気を引き起こす」と捉えられないような記載にする

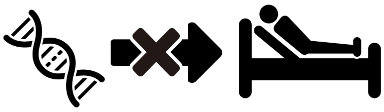

全体を通じて

01 個人情報を守る

患者・研究参加者の個人情報が特定されないような記載にする

希少疾患の場合は、個人特定に強い留意が必要である。一方で、報道によって、患者・研究参加者に**リスクを上回るメリット**が生じる可能性にも留意する。

Note

- 希少疾患については、罹患者数が少ないために個人特定性が高くなり、また、関係する読者が限定されるという性質もあることから、記事化を見送る判断もメディア側にはあり得る。しかし逆に、埋もれがちなことをきちんと伝えて光を当てる必要がある、という使命もあるので、その間のバランスをとる必要が生じる。

- ゲノム情報は、一定の条件を満たすと法的に個人情報として扱われる[*1]。
しかし、その条件の詳細を知る人は、研究者にもメディアにも少ない。発信・報道の際に最新の状況を確認する必要がある。

*1）個人情報保護委員会『個人情報の保護に関する法律についてのガイドライン（通則編）2-2 個人識別符号 イ』を参照
https://www.ppc.go.jp/personalinfo/legal/guidelines_tsusoku/

For Media

- 記事化にあたって、関係者を取材するなどしてそれを反映した結果、意図せず個人特定性が高い記載になることにも留意が必要。

02 不適切な対応等を防ぐ

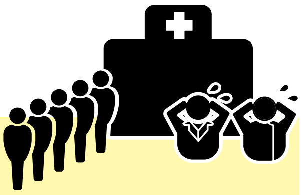

読んだ人* が不適切な対応を行うことがないような記載にする

*1：**患者・研究参加者**が、自らの遺伝性疾患のリスクを知り、それに対処するために不適切な対応を行ってしまわないような記載にする。

*2：**家族や周辺の人**が、関係が近い人の遺伝性疾患のリスクを知り、不適切な対応を推奨してしまわないような記載にする。

*3：**医療者**が、患者に対して、（報道等から入手した）知見による理解に基づいて、不適切な対応を行ってしまわないような記載にする。

不適切な対応には、医学的には推奨されない頻回の検査受診などの過剰な対応や、受診や予防接種等を控えることなどが考えられる。患者・研究参加者等が自ら選んで積極的に起こす行動のみならず、何もしないことなども対応に含まれる。

Note

- [ ] 行動変容が明らかに望まれる状況（例：禁煙）においても、その推奨を成果そのものから導き出すことができない場合には、記載を考慮する必要がある。

For Media

- [ ] 報道の結果として、対象となる人々に研究成果と不釣り合いな期待・不安・恐怖を起こさせることに対して留意が必要である。

03

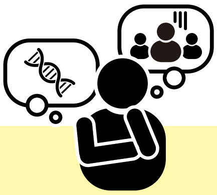

関係者に配慮する

患者・研究参加者自身が、またはその親族や近しい人々がお互いに対して、負い目を感じないような記載にする

科学的な限界など、**留保**（断定的な事実ではなく、今回出された一つの成果であることなど）を明確に伝えること。

Note

- 関係者への衝撃を恐れるあまりに、科学的事実の隠蔽にならないようにすること。
  例１）ミトコンドリア遺伝子の変異による疾患の場合に、母から子に遺伝する、ということを避けて記載する
  例２）特定の遺伝子に関係する疾患を記載する場合に、影響が大きそうな疾患名を意図的に除外する

- 専門用語の中には、当事者に衝撃を与えるような種類の言葉があることが想定される（例：創始者効果、創始者バリアント）。こうした場合には、プレスリリース等でその言葉を使わないようにするか、より丁寧な説明を行うようにすること。

For Media

- 報道する価値が高い事案で関係者に負の感情を喚起させる恐れがある場合には、より慎重な検討を行うこと。

04 偏見・差別を防ぐ

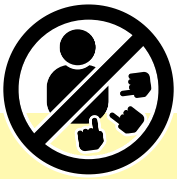

特定の地域や集団への偏見や差別につながらないような記載にする

特定の地域や集団には、**居住地域**や**出身地**、**祖先性**のみならず、**文化的な背景**や**嗜好**なども含まれる。

Note

- 地域による遺伝的な「差」の存在そのものが、その地域に対する差別として受け止められた事例もある。

- 居住地域、祖先性、民族などと遺伝の関係は極めてセンシティブであり、米国の科学アカデミーからそれをテーマにした報告書[*1]がまとめられている。特に「人種（race）」という言葉は、その言葉自体を避ける情勢になっている。我が国において、社会的にどのようにコンセンサスをまとめていくか、現在の課題となっている。

  *1) Using Population Descriptors in Genetics and Genomics Research: A New Framework for an Evolving Field (2023), National Academies of Sciences, Engineering, and Medicine
  https://nap.nationalacademies.org/catalog/26902/using-population-descriptors-in-genetics-and-genomics-research-a-new

- プレスリリース・報道において、差別・偏見を慎重に避けたとしても、それを受けて悪用される可能性がある。悪用に対する対処・予防的措置なども考慮・検討が必要。

For Media

- 差別・偏見が生じてしまう可能性への恐れよりも報道することの価値が優先されると考えられる場合には、そのことを慎重に検討した上で報道する。

05 遺伝子決定論を避ける

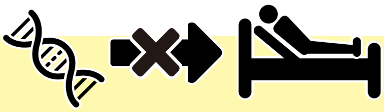

ゲノム・遺伝子と病気との関係について、「特定の違いがあれば必ず病気を引き起こす」と捉えられないような記載にする

遺伝子配列の違いが、全て親子で**遺伝する性質のものとは限らない**ことに注意して記載すること。体細胞変異と生殖細胞系列の違いにも留意すること。

Note

- ゲノムと病気の関係性を取り上げる際には、専門用語の扱いと、それが報道時にどの言葉に置き換えられて伝えられるかに留意する。
例）変異、発症リスク、遺伝（heredity）と遺伝子（gene）

For Media

- 特に疫学研究の場合に、導き出せる結果は相関関係に留まることが多い。わかりやすく記載しようとした結果、因果関係のように読める記載になりやすいことに留意する。
（P.4 Ⅴ参照）

～全体を通じて～

- 本ガイドの順守を検討することで、発信意欲が減退したり、発信に慎重になるあまりに**科学的事実等を歪曲・隠蔽することにつながらないように留意が必要**である。

- **発信済みのプレスリリースの一部が誤った解釈をもとに拡散される可能性**がある。こうしたリスクを事前に予想してあらかじめ対策を施したり、事後にウェブ等で追加の情報発信を行うことを検討する。

- 研究成果の解釈について、**誤解を防ぐための留意点がある場合には、明示する**ことが望ましい。

- 遺伝性疾患について伝える際には、疾患の原因のみならず、**対処法についての言及があることが望ましい**という意見がある。プレスリリースの成果と直接の関係がないことを明示した上で、記載が可能であるか、**プレスリリース発信側・メディア側双方で検討**することが望ましい。

- 「病的バリアントを持つとリスクが高まる」等の話題のときには、「**そのバリアントを持たなくても対象の疾患になる人はいる（また、その逆もある）**」ということが明示的に記載されることが望ましい。

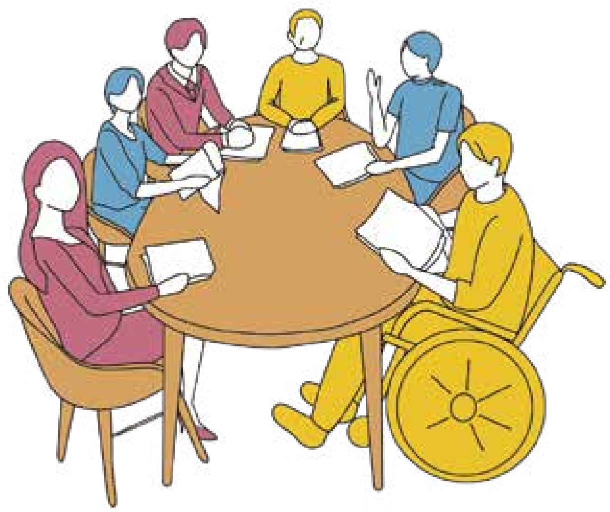

参考文献

1. Ascencio-Carbajal T, Saruwatari-Zavala G, Navarro-Garcia F, Frixione E.
Genetic/genomic testing: defining the parameters for ethical, legal and social implications (ELSI). BMC Med Ethics. 2021 Nov 23;22(1):156.

2. Caulfield T, Chandrasekharan S, Joly Y, Cook-Deegan R. Harm, hype and evidence:ELSI research and policy guidance. Genome Med. 2013 Mar 26;5(3):21.

3. Burke W, Diekema DS. Ethical issues arising from the participation of children in genetic research. J Pediatr. 2006 Jul;149(1 Suppl):S34-8.

4. WMA DECLARATION OF REYKJAVIK – ETHICAL CONSIDERATIONS REGARDING THE USE OF GENETICS IN HEALTH CARE. 2019.

5. 医学系研究をわかりやすく伝えるための手引き【第1版】2022、【第2版】2023
https://ez2understand.ifi.u-tokyo.ac.jp/common/pdf/guidebook.pdf

6. 医療情報をわかりやすく発信するプロジェクト
https://ez2understand.ifi.u-Tokyo.ac.jp/

7. 日本神経科学学会 科学コミュニケーション･ガイドライン 研究成果のプレスリリース
https://www.jnss.org/hp_images/images/Science%20Communication%20Guidelines.pdf

8. 日本心理学会　科学コミュニケーション･ガイドライン 研究成果のプレスリリース
https://psych.or.jp/wp-content/uploads/2020/04/SC_guideline.pdf

9. Stempra Guide To Being a Media Officer 2019
https://stempra.org.uk/wp-content/uploads/2019/03/1902.21_Officers-Press-Guide-UPDATE_A4_WebOfficePrinter.pdf

ゲノム医療・研究のプレスリリース発出ガイド

**発行年月**	2025年2月（第2版）
**発行**	ゲノム医療・研究推進社会に向けた試料・情報の利活用とPPI施策に関する研究開発（代表：吉田雅幸 東京科学大学教授）
**分担研究開発課題名**	ゲノム医療・研究にかかるコミュニケーション方策の向上<br>（研究開発分担者：長神風二 東北大学東北メディカル・メガバンク機構教授）
**研究費**	国立研究開発法人日本医療研究開発機構（AMED）<br>ゲノム医療実現バイオバンク利活用プログラム令和4年度採択課題
**制作者**	長神風二、荒川美咲
**制作協力**	ゲノム医療・研究推進社会に向けた試料・情報の利活用とPPI施策に関する研究開発研究分担者・研究協力者、PPI委員のみなさま、インタビューにご協力いただいたみなさま
**連絡先**	**東北大学東北メディカル・メガバンク機構広報・企画部門**<br>980-8573<br>宮城県仙台市青葉区星陵町2-1<br>022-717-7908
**本研究に関するウェブサイト**	https://genomeppi.jp/
